# Properties of Soy Protein Isolate Biopolymer Film Modified by Graphene

**DOI:** 10.3390/polym9080312

**Published:** 2017-07-27

**Authors:** Yufei Han, Kuang Li, Hui Chen, Jianzhang Li

**Affiliations:** 1 Key Laboratory of Wood Material Science and Utilization (Beijing Forestry University), Ministry of Education, Beijing 100083, China; hanyufei@bjfu.edu.cn (Y.H.); kuangli@bjfu.edu.cn (K.L.); 2 Beijing Key Laboratory of Wood Science and Engineering, Beijing Forestry University, Beijing 100083, China; 3 College of Materials Science and Technology, Beijing Forestry University, Beijing 100083, China

**Keywords:** soy protein isolate, graphene, mechanical property, water resistance

## Abstract

This study applied a facile and green approach to synthesize a stable graphene aqueous dispersion, and the graphene aqueous dispersion was employed to modify the renewable, compatible and biodegradable soy-protein-isolated (SPI) films to enhance their thermal stability, mechanical properties and water resistance. Atomic force microscopy (AFM) images confirmed the monolayer structure of graphene. The hydrogen bonds and π–π interactions between graphene and the SPI molecules were showed with the attenuated total reflectance Fourier transform infrared (ATR FT-IR) spectroscopy, and X-ray diffraction (XRD). As expected, compared to the pure SPI film, the tensile strength (*TS*) of the film with 74% graphene increased by 27.22% and the total soluble matter (TSM) of the film with 93% graphene decreased by 11.30%.

## 1. Introduction

Nowadays, with the increased awareness of sustainable development and environmental protection, interest has risen in developing biopolymer films as an environment friendly and renewable alternative to petroleum-based materials [1]. These films are mainly from natural polymers, such as protein, lipid biopolymer and cellulose, which offer the advantages of sustainability, biodegradability and biocompatibility [2].

Among the biopolymer studies, soy protein isolate (SPI), a common by-product of the edible oil industry, showed a wide range of potential applications in drug delivery, packaging and mulching fields due to its low cost, abundance, biocompatibility, sustainability and film-forming capacity [3,4,5]. However, the drawbacks of SPI films, such as poor mechanical strength and high water sensitivity have limited their applications [6,7]. Therefore, many efforts have been made to enhance their performance via physical methods, enzymatic treatments and chemical cross-linking approaches [8].

Recently, graphene has attracted increasing interest as an emerging functional material. Graphene is a two-dimensional (2D) crystalline monolayer sheet, with sp^2^-hybridized carbon atoms arranged in a honeycomb lattice [9,10,11]. The outstanding thermal, mechanical, electrical and hydrophobic properties offered broad applications in biomedicine, reinforced composites, catalytic, conductive materials, and energy resources [9,10,12,13]. The reported methods of synthesizing graphene were mainly exfoliation, epitaxial growth, and chemical vapor deposition (CVD) [14,15,16]. However, these methods have the disadvantages of toxicity, a lack of compatibility and the forming of additional defects. Therefore, a facile and green approach for preparing stable graphene aqueous dispersion was required.

Inspired by the excellent hydrophobic and mechanical properties of graphene, Wang et al., synthesized graphene-reinforced poly(vinyl alcohol) (PVA) composite films [17]. The results showed that the tensile strength increased by 212% and elongation increased by 34% with only 0.5 wt % graphene content. The water absorption ratio of graphene–PVA composite films decreased from 105.2 to 48.8%. Jiang et al. explained that the enhanced performance was due to the hydrogen bonds between the PVA and the graphene molecules [18]. The graphene–nylon nanocomposites also exhibited obvious improvements in mechanical properties and water resistance with very low filler loading [19]. Zhang et al. fabricated ultra-strong, ultra-tough, and highly conductive rGO (reduced graphene oxide) films based on the hydrogel casting technique, demonstrating high performance through the synergistic effect from π–π interactions and hydrogen bonds [20]. These previous studies indicated that graphene can achieve better enhancement effects due to the interactions between graphene and the polymer matrix. 

In this study, we compounded series of graphene with different ultrasonic time via a facile method, and prepared renewable and environment friendly SPI–graphene composite films. The hydrogen bonds and π–π interactions between the SPI and graphene molecules improved the mechanical properties, thermal stability and water resistance of the resultant composite films. This advanced performance provides SPI–graphene films wide potential application in drug delivery, packaging and the food industry.

## 2. Materials and Methods 

### 2.1. Materials

SPI with a protein content of 95% was purchased from Yuwang Ecological Food Industry Co., Ltd. (Yucheng, China). Graphite powder was provided by Sinopharm Chemical Reagent Co., Ltd. (Shanghai, China). Bovine serum albumin of biotechnology grade was obtained from Beijing Labest Bio Technology Co., Ltd. (Beijing, China). Glycerol (99% purity) of biotechnology grade, sodium hydroxide of analytical grade and other chemical reactants of analytical grade were purchased from Beijing Chemical Reagents (Beijing, China).

### 2.2. Synthesis of Aqueous Graphene Dispersion

50.0 mg of bovine serum albumin (BSA) powder was mixed with 900 mL of deionized water at 50 °C and stirred for 12 h. The pH of the aqueous BSA solution was adjusted to 3.6 with dilute HCl solution. Then 1 g graphite power were dispersed in 600 mL of the aqueous BSA solution. The mixture was probe-sonicated and stirred for 6, 12, 18, 24, 30 and 36 h. The product was allowed to stand for 24 h at room temperature to settle some of the unreacted graphite particles and large graphene aggregates. It was then centrifuged at 3000 rpm for 30 min to separate the supernatant from the sediment. The concentration of aqueous graphene dispersion was calculated from the Beer–Lambert law with the extinction coefficient of graphene (α = 1390 mL·mg^−1^·m^−1^) previously determined [21] at the wavelength of the highest absorbance peak (269 nm) in ultraviolet-visible absorption spectra.

### 2.3. Prepare of Graphene–SPI Films

The SPI film-forming solutions were prepared by SPI (4.0 g), glycerol (2.0 g), graphene aqueous dispersion and deionized water. The pH of the mixture was adjusted to 9.0 ± 0.1 with sodium hydroxide solution, then heated in a water bath at 85 °C for 30 min. The solution (75.0 g) was poured onto a Teflon-coated plate after removing bubbles by ultrasound treatment. The films were dried in drying oven at 45 °C for 24 h, and preconditioned at the relative humidity of 50 ± 2% and temperature of 25 ± 2 °C for 48 h before testing. The composition parts of the films were presented in Table 1. The graphene content was calculated by the proportion of graphene aqueous dispersion in the entire solution system, and applied to the whole experiment.

### 2.4. Characterization of Graphene Aqueous Dispersion and SPI–Graphene Films

The TU-1901 ultraviolet-visible spectrophotometer (UV–vis, Beijing Purkinje General, Beijing, China) was used to record the absorption spectra of aqueous graphene within the range of 250–400 nm.

The morphology of graphene was observed by atomic force microscopy (AFM, Bruker Multimode 8, Billerica, MA, USA). The topographic (height) and phase images were collected in the tapping mode using a monolithic Si tip with a resonance frequency between 250 and 300 kHz.

The surface morphology of the graphene–SPI films was observed using the SU8010 field emission scanning electron microscopy (SEM, Hitachi Ltd., Tokyo, Japan) with an acceleration voltage of 5 kV.

The chemical structure of the graphene–SPI films was examined according to the Nicolet 6700 attenuated total reflectance Fourier transform infrared spectroscopy (ATR FT-IR, Thermo Scientific, Pittsburgh, PA, USA) with a wave number range from 650 to 4000 cm^−1^. A total of 32 scans were performed at 4 cm^−1^ resolution.

Samples were dried at 45 °C for 24 h before testing. The thermal stability of the graphene–SPI films was measured using the Q50 thermo gravimetric analysis (TGA) analyzer (TA Instrument, New Castle, DE, USA) with a rate of 10 °C·min^−1^. The temperature ranged from 30 to 600 °C under a nitrogen atmosphere (100 mL·min^−1^) to avoid thermo-oxidative reactions.

X-ray diffraction (XRD) patterns were observed by the D8 advance diffractometer (Bruker, Billerica, MA, USA) equipped with a Cu Kα radiation source. The 2θ value was ranged from 5° to 60° in continuous scanning mode with an increment of 0.02°.

The surface hydrophilicity of the graphene–SPI films was investigated using the water contact angles (WCA, OCA-20 Dataphysics Instruments GmbH, Filderstadt, Germany). A sessile droplet (3 μL, measured by microsyringe) of distilled water was dropped onto the surface of the films. The angles of both sides were recorded at an interval from 0.1 to 180 s. Five replicates were conducted for each specimen.

### 2.5. Mechanical Properties

Each sample was cut into 10 × 80 mm pieces and the thickness of the film was measured with a digital micrometer (0–25 ± 0.001 mm) before testing. The mechanical properties were determined on the tensile testing machine (WDW3020, Changchun Kexin Instrument Co., Ltd., Changchun, China) according to ISO527-3:1995 (E). The tensile strength (*TS*), elongation at break (*EB*) and Young’s modulus (*E*) were determined by five replicates of each film.

### 2.6. Water Barrier Properties

Three specimens (20 × 20 mm^2^) of each film were tested to determine the moisture content (*MC*), total solution matter (*TSM*) and water uptake (*WU*).

The initial mass (*m*_a_) was determined. The specimens were dried in an air-circulating oven at 103 ± 2 °C for 24 h and weighed again (*m*_b_). *MC* was calculated as follows:

*MC* (%) = (*m*_a_ − *m*_b_)/*m*_a_ × 100 (1)

Afterward, specimens were immersed in a breaker containing 30 mL of deionized water for 24 h at room temperature. Sodium azide was also added to the breaker to inhibit microbial growth. The insoluble matter was separated and dried in an air-circulating oven at 103 ± 2 °C for 24 h again. The mass of the specimens was marked as *m*_c_. *TSM* was calculated as follows:

*TSM* (%) = (*m*_b_ − *m*_c_)/*m*_b_ × 100 (2)

The specimens were firstly stored in P_2_O_5_-regulated desiccators (0% relative humidity) for 48 h, and the initial mass was weighed as *m*_d_. The films were immersed in a breaker with 30 mL of deionized water at room temperature for 24 h. Then the water was moved and the mass of specimens were weighted as *m*_e_. *WU* was calculated as follows:

*WU* (%) = (*m*_e_ − *m*_d_)/*m*_d_ × 100 (3)

## 3. Results

### 3.1. Characterization of Graphene Aqueous Dispersion

**Figure 1:**
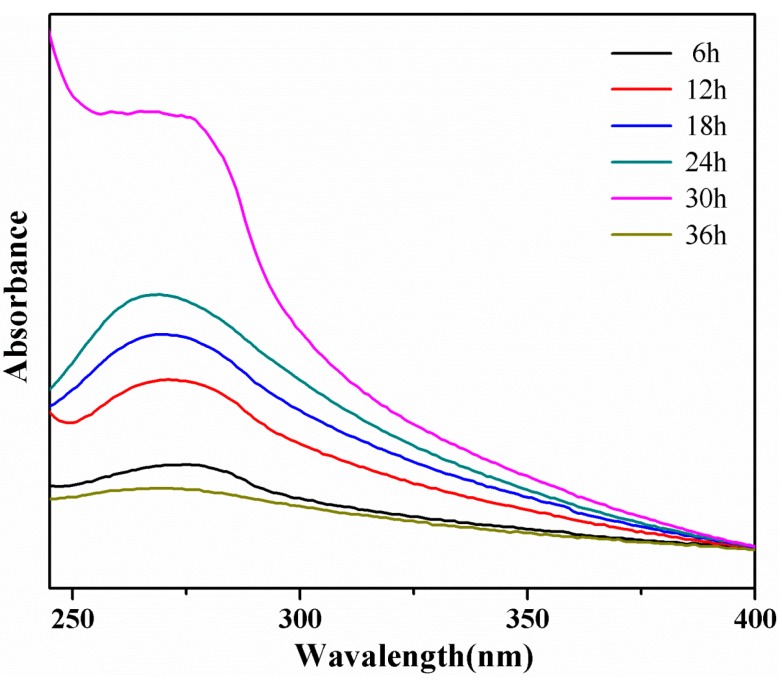
UV–vis absorption spectra of graphene with ultrasonic time.

UV–vis spectroscopy was used to confirm the presence of graphene. Figure 1 shows the UV–vis absorption spectra of graphene with different ultrasonic times of 6, 12, 18, 24, 30 and 36 h. The shapes of different spectra were similar, except the ultrasonic time of 30 h, which may due to the excessive ultraviolet terminal absorption. Graphene has a broad absorption spectrum of 269 nm^−1^ [22,23]. The highest absorbance peak appeared when the ultrasonic time was 24 h and the graphene concentration was 0.049 mg/mL calculated by the Beer–Lambert law. An obvious blue shift occurred with the increase of the ultrasonic time. It demonstrated that the graphene was prepared successfully.

**Figure 2:**
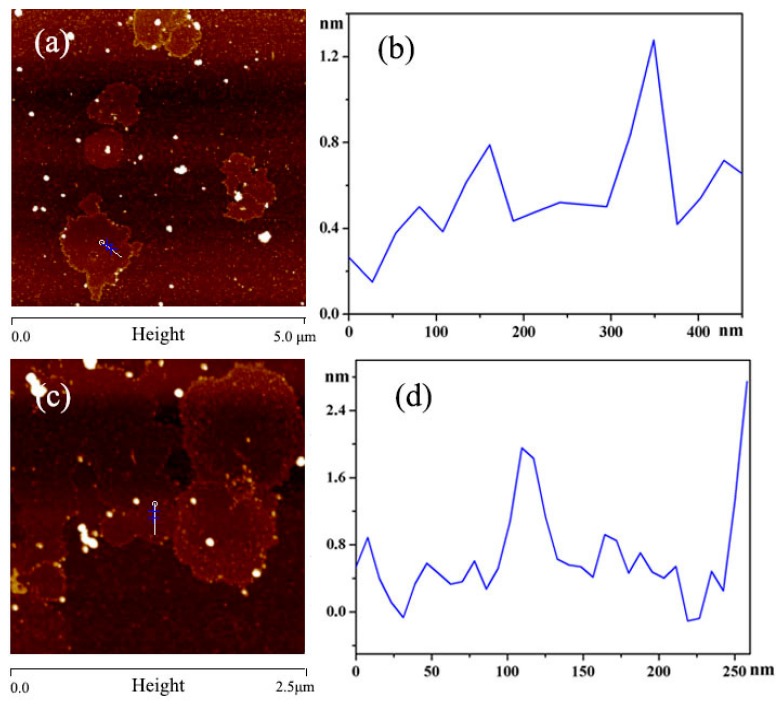
AFM images of graphene: (**a**,**c**) 2D morphologies of graphene with different magnifications; (**b**,**d**) height images of graphene with different magnifications.

The stripped graphene aqueous dispersion (1 mL) was obtained, then diluted five times directly before investigated the surface morphology of the graphene by AFM. The 2D morphologies and height images are shown in Figure 2. It was obvious that the 2D morphologies exhibitd the shape of monolayer in Figure 2a,c [24], and the white spherical structure was BSA. The height images indicated that the thickness of the graphene layer was about 1 nm in Figure 2b,d. These results demonstrated that the graphene was prepared successfully, which was consistent with the results of the UV–vis.

### 3.2. Structural Analysis of SPI-Based Films

**Figure 3:**
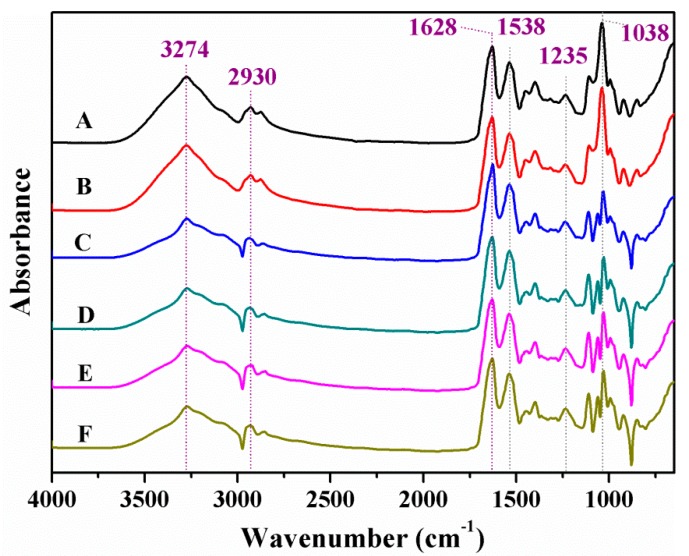
ATR FT-IR spectra of different SPI-based films.

ATR FT-IR was used to investigate the structural characteristics of the SPI-based films (Figure 3). These spectra showed similar absorption peaks in the range of 4000 to 600 cm^−1^. The increasing intensity of the broad absorption band at 3274 cm^−1^ was attributed to the O–H stretching in the SPI films [6]. The peaks at 2930 cm^−1^ were the stretching vibrations of the methylene groups [25]. Among all the SPI-based films, the peaks at 1628, 1538, 1235 cm^−1^ were the main characteristic amide bonds of amide І (C=O stretching), amide ІІ (N–H bending), and amide ІІІ (C–N and N–H stretching), respectively [26,27]. The band at 1038 cm^−1^ corresponded to the hydrogen bond between SPI and the graphene molecules. Based on the above analysis, we concluded that the interactions between SPI and the graphene molecules were mainly due to the hydrogen bonds, and the π–π interactions also provided a possibility for the modification of SPI molecules.

**Figure 4:**
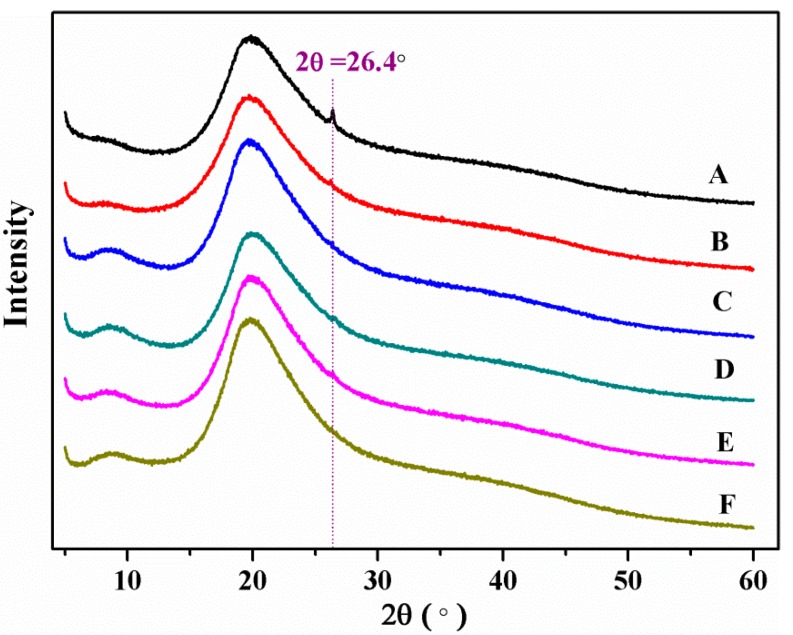
XRD patterns of different SPI-based films.

The structure of SPI-based films was observed by XRD (Figure 4). Clearly, the XRD patterns of these SPI-based films were similar. The two wide diffraction peaks at 2θ = 9.1° and 20° belonged to the SPI secondary conformation—α-helix and β-sheet, respectively. Furthermore, the intensity of the peaks at 2θ = 9.1° and 20° were decreased due to the increased amount of graphene [11]. The result indicated that the addition of graphene transformed the conformation of the soy protein molecules and destroyed the regular arrangement of the SPI molecular chains. The diffraction peak at 2θ = 26.4° corresponded to graphene, which showed good compatibility between graphene and the SPI matrix.

### 3.3. Micromorphology of SPI-Based Films

**Figure 5:**
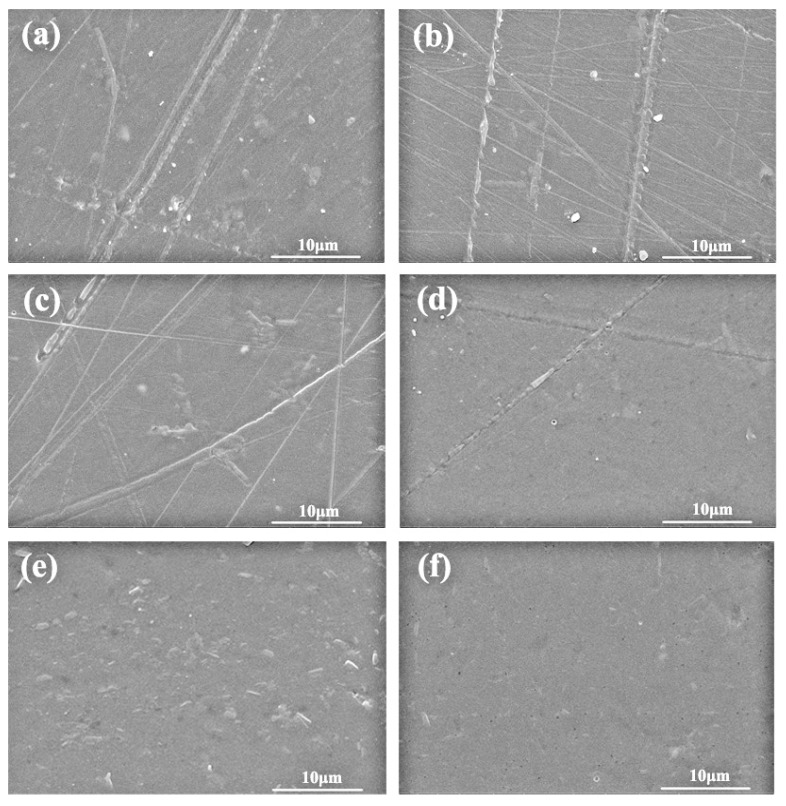
SEM images of SPI-based films: (**a**) 93% graphene aqueous dispersion; (**b**) 74% graphene aqueous dispersion; (**c**) 56% graphene aqueous dispersion; (**d**) 37% graphene aqueous dispersion; (**e**) 19% graphene aqueous dispersion; (**f**) the pure SPI film.

The surface morphology of graphene–SPI films was observed by SEM (Figure 5). Obviously, Figure 5a–d shows the surface morphology with straight line-like shapes on the modified SPI-based films, compared with the smoother surface of the pure SPI film in Figure 5f [28]. The results indicated that graphene was completely compatible with the SPI matrix, and that the introduction of the graphene changed the crystal structures of the SPI molecules and increased the regularity of the molecules’ arrangement.

### 3.4. Thermal Stability of SPI-Based Films

**Figure 6:**
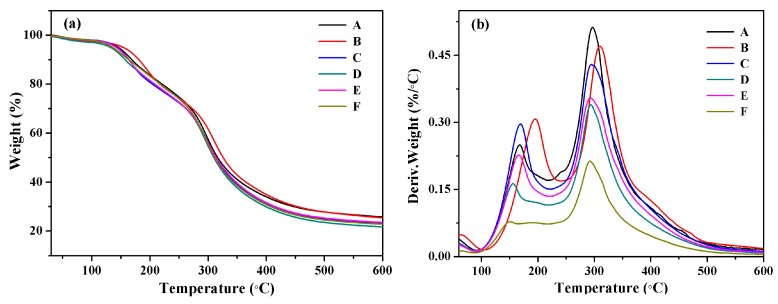
(**a**) The thermo gravimetric (TG) and (**b**) derivative TG (DTG) curves of SPI-based films.

The thermal properties of these SPI-based films were investigated by thermos-gravimetric (TG) and derivative TG (DTG) analysis, as shown in Figure 6. The thermal degradation data were summarized in Table 2. The initial degradation stage from 40 to 100 °C was the dehydration reaction. The second stage from 100 to 250 °C was the degradation of glycerol. The last stage from 250 to 400 °C was mainly related to the breaking of the hydrogen bond and the degradation of backbone peptides [29,30,31]. The addition of graphene improved the maximum degradation temperature. Compared to the pure SPI film (F), the temperature at the maximal degradation rate (*T*_max1,2_) of sample B (with 74% graphene content) increased from 151 to 195 °C, and from 293 to 309 °C. Overall, the improved thermal stability was attributed to the hydrogen bonds and π–π interactions between graphene and the SPI matrix. However, the excessive content of graphene may have strengthened the π–π interactions between graphene molecules, thereby weakening the interactions between the graphene and the SPI molecules.

### 3.5. Mechanical Properties and Water Resistance

**Figure 7:**
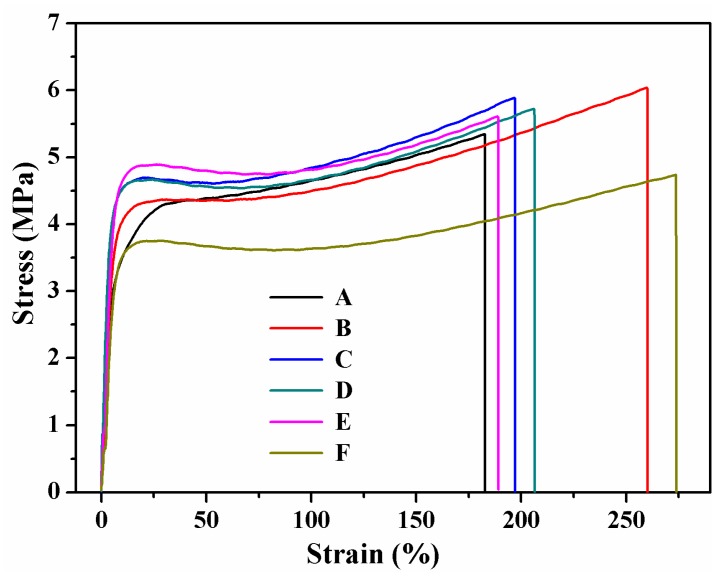
Stress–strain curves of different SPI-based films.

The results of tensile strength (*TS*), Young’s modulus (*E*) and elongation at break (*EB*) of SPI-based films are shown in Figure 7 and Table 3. The stress–strain curves in Figure 7 show that the SPI-based films experienced stretching deformation and fracture failure after the application of a certain amount of load. The addition of graphene improved the *TS* and *E*, while reducing the *EB*. Compared to the pure SPI film, the *TS* of sample B (with 74% graphene content) increased from 4.74 to 6.03 Mpa, accounting for the increase of 27.22%. The results may be attributed to the deformation of SPI molecule chains. Graphene was distributed in the SPI matrix in the form of a single layer, which enhanced interaction and prevented slipping [32,33,34]. However, the excessive introduction of graphene may have strengthened the π–π interactions between graphene molecules, thereby weakening the interaction between the graphene and the SPI molecules. 

**Figure 8:**
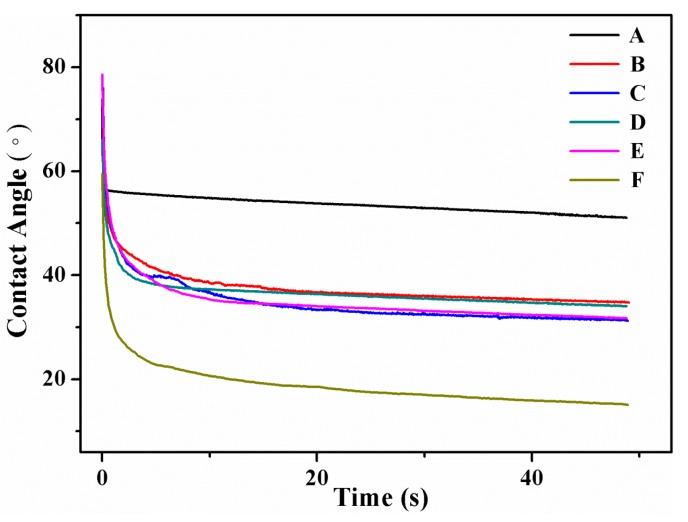
Water contact angle of different SPI-based films.

The water contact angles of SPI-based films are shown in Figure 8, indicating the surface hydrophobia of the SPI-based films. As can be seen, compared to the pure SPI film (F), the contact angle of the modified SPI films increased obviously, which may be interpreted as the interaction between graphene and the SPI molecules exposed more hydrophobic groups, thereby exhibiting improved surface hydrophobic properties [4,35].

Table 4 showed the moisture content (*MC*), total soluble matter (*TSM*) and water uptake (*WU*) of the six groups of SPI-based film samples. The introduction of graphene did not result in significant changes in the *MC* result. The addition of graphene decreased the *TSM* and *WU*, but the *WU* trend was not obvious. Compared to the pure SPI film, the *TSM* of sample A (with 93% graphene content) decreased by 11.30%. The results may be accounted as the outstanding compatibility and strong interactions between graphene and the SPI matrix, which exposed more hydrophobic groups, thereby exhibiting improved surface hydrophobic properties [36,37,38].

## 4. Conclusions

In the study, we synthesized graphene aqueous dispersion via a simple and green method successfully. Composite SPI–graphene films with enhanced thermal stability, mechanical properties and water resistance were prepared. When preparing the graphene aqueous dispersion, the best ultrasonic time was 24 h. Compared to the pure SPI film, the *TS* of sample B (with 74% graphene content) increased by 27.22%. Water resistance and thermal abilities were also improved due to the addition of graphene. This improved performance may be due to the hydrogen bonds and π–π interactions between graphene and the SPI matrix, which were confirmed by the ATR FT-IR and XRD. Overall, this research indicated that SPI–graphene composite films have potential applications in drug delivery, packaging and mulching.

## Figures and Tables

**Table 1:** The composition parts of graphene–SPI films.

Sample	SPI (g)	Glycerol (g)	Water (g)	Graphene Aqueous Dispersion (mL)	Graphene Content (%)
A	4.0	2.0	0	75.0	93.0
B	4.0	2.0	15.0	60.0	74.0
C	4.0	2.0	30.0	45.0	56.0
D	4.0	2.0	45.0	30.0	37.0
E	4.0	2.0	60.0	15.0	19.0
F	4.0	2.0	75.0	0	0

**Table 2:** TG parameters for the thermal degradation of SPI-based films.

Sample	*T*_max1_^a^ (°C)	*T*_max2_^a^ (°C)
A	167	297
B	195	309
C	169	298
D	156	294
E	166	292
F	151	293

^a^ Peak temperature.

**Table 3:** The thickness, tensile strength (*TS*), Young’s modulus (*E*) and elongation at break (*EB*) of different SPI-based films.

Sample	Thickness (mm)	*TS* (MPa)	*E* (MPa)	*EB* (%)
A	0.32 (0.028) ^a^	5.35 (0.08)	71.29 (6.97)	182.84 (4.58)
B	0.32 (0.025)	6.03 (0.29)	113.03 (0.26)	260.13 (6.69)
C	0.29 (0.010)	5.88 (0.17)	104.99 (5.91)	197.07 (2.54)
D	0.31 (0.011)	5.63 (0.37)	89.84 (2.53)	206.01 (4.83)
E	0.29 (0.015)	5.60 (0.25)	58.34 (4.25)	189.09 (4.58)
F	0.29 (0.035)	4.74 (0.18)	47.58 (4.45)	273.91 (1.84)

^a^ Mean (standard deviation).

**Table 4:** The moisture content (*MC*), total soluble matter (*TSM*) and water uptake of different SPI-based films.

Sample	*MC* (%)	*TSM* (%)	*WU* (%)
A	20.84 (0.14) ^a^	29.60 (0.09)	190.74 (4.40)
B	21.98 (0.15)	30.14 (0.25)	216.29 (2.22)
C	19.75 (0.48)	31.84 (0.18)	194.14 (4.81)
D	19.90 (0.40)	31.95 (0.17)	221.64 (1.22)
E	20.70 (0.03)	31.40 (0.52)	201.97 (5.93)
F	20.18 (0.02)	33.37 (0.86)	261.79 (2.71)

^a^ Mean (standard deviation).

## References

[1] Koshy R.R., Mary S.K., Thomas S., Pothan L.A. (2015). Environment friendly green composites based on soy protein isolate—A review. Food Hydrocoll..

[2] Ciannamea E.M., Stefani P.M., Ruseckaite R.A. (2014). Physical and mechanical properties of compression molded and solution casting soybean protein concentrate based films. Food Hydrocoll..

[3] Tansaz S., Boccaccini A.R. (2016). Biomedical applications of soy protein: A brief overview. J. Biomed. Mater. Res. A.

[4] Galus S., Mathieu H., Lenart A., Debeaufort F. (2012). Effect of modified starch or maltodextrin incorporation on the barrier and mechanical properties, moisture sensitivity and appearance of soy protein isolate-based edible films. Innov. Food Sci. Emerg. Technol..

[5] Dash S., Swain S.K. (2013). Effect of nanoboron nitride on the physical and chemical properties of soy protein. Compos. Sci. Technol..

[6] González A., Strumia M.C., Alvarez Igarzabal C.I. (2011). Cross-linked soy protein as material for biodegradable films: Synthesis, characterization and biodegradation. J. Food Eng..

[7] Song X., Zhou C., Fu F., Chen Z., Wu Q. (2013). Effect of high-pressure homogenization on particle size and film properties of soy protein isolate. Ind. Crops Prod..

[8] Xing F., Zhang S., Li J., Li L., Shi J. (2016). Crosslinked chitosan-based biocomposite films modified with soy protein isolate. Polym. Compos..

[9] Rao C.N.R., Sood A.K., Subrahmanyam K.S., Govindaraj A. (2009). Graphene: The new two-dimensional nanomaterial. Angew. Chem. Int. Ed..

[10] Kuilla T., Bhadra S., Yao D.H., Kim N.H., Bose S., Lee J.H. (2010). Recent advances in graphene based polymer composites. Prog. Polym. Sci..

[11] Sun H., Ge G., Zhu J., Yan H., Lu Y., Wu Y., Wan J., Han M., Luo Y. (2015). High electrical conductivity of graphene-based transparent conductive films with silver nanocomposites. RSC Adv..

[12] Stankovich S., Dikin D.A., Dommett G.H.B., Kohlhaas K.M., Zimney E.J., Stach E.A., Piner R.D., Nguyen S.T., Ruoff R.S. (2006). Graphene-based composite materials. Nature.

[13] Lee C., Wei X.D., Kysar J.W., Hone J. (2008). Measurement of the elastic properties and intrinsic strength of monolayer graphene. Science.

[14] Whitener K.E., Sheehan P.E. (2014). Graphene synthesis. Diam. Relat. Mater..

[15] Shams S.S., Zhang R., Zhu J. (2015). Graphene synthesis: A review. Mater. Sci. Pol..

[16] Gurunathan S., Han J., Kim J.H. (2013). Humanin: A novel functional molecule for the green synthesis of graphene. Colloids Surf. B Biointerfaces.

[17] Wang J., Wang X., Xu C., Zhang M., Shang X. (2011). Preparation of graphene/poly(vinyl alcohol) nanocomposites with enhanced mechanical properties and water resistance. Polym. Int..

[18] Zhang Y., Gong S., Zhang Q., Ming P., Wan S., Peng J., Jiang L., Cheng Q. (2016). Graphene-based artificial nacre nanocomposites. Chem. Soc. Rev..

[19] Jin J., Rafiq R., Gill Y.Q., Song M. (2013). Preparation and characterization of high performance of graphene/nylon nanocomposites. Eur. Polym. J..

[20] Zhang M., Huang L., Chen J., Li C., Shi G. (2014). Ultratough, ultrastrong, and highly conductive graphene films with arbitrary sizes. Adv. Mater..

[21] Guardia L., Fernández-Merino M.J., Paredes J.I., Solís-Fernández P., Villar-Rodil S., Martínez-Alonso A., Tascón J.M.D. (2011). High-throughput production of pristine graphene in an aqueous dispersion assisted by non-ionic surfactants. Carbon.

[22] Huang L.Y., Lu C.X., Wang F., Dong X.Z. (2016). Piezoelectric property of pvdf/graphene composite films using 1H, 1H, 2H, 2H-perfluorooctyltriethoxysilane as a modifying agent. J. Alloys Compd..

[23] Hajian M., Reisi M.R., Koohmareh G.A., Zanjani Jam A.R. (2012). Preparation and characterization of polyvinylbutyral/graphene nanocomposite. J. Polym. Res..

[24] Mishchenko A., Eckmann A., Grigorieva I.V., Novoselov K.S. (2016). Fluorination Clusters on Graphene Resolved by Conductive AFM.

[25] Pan H., Jiang B., Chen J., Jin Z. (2014). Blend-modification of soy protein/lauric acid edible films using polysaccharides. Food Chem..

[26] González A., Alvarez Igarzabal C.I. (2013). Soy protein—Poly (lactic acid) bilayer films as biodegradable material for active food packaging. Food Hydrocoll..

[27] Mauri A.N., Añón M.C. (2006). Effect of solution ph on solubility and some structural properties of soybean protein isolate films. J. Sci. Food Agric..

[28] Ahn H.S., Kim H., Kim J.M., Park S.C., Kim J.M., Kim J., Kim M.H. (2013). Controllable pore size of three dimensional self-assembled foam-like graphene and its wettability. Carbon.

[29] Kumar R., Anandjiwala R.D., Kumar A. (2015). Thermal and mechanical properties of mandelic acid-incorporated soy protein films. J. Ther. Anal. Calorim..

[30] González A., Alvarez Igarzabal C.I. (2015). Nanocrystal-reinforced soy protein films and their application as active packaging. Food Hydrocoll..

[31] Swain S.K., Priyadarshini P.P., Patra S.K. (2012). Soy protein/clay bionanocomposites as ideal packaging materials. Polym. Plast. Technol. Eng..

[32] Kang H., Wang Z., Zhang W., Li J., Zhang S. (2016). Physico-chemical properties improvement of soy protein isolate films through caffeic acid incorporation and tri-functional aziridine hybridization. Food Hydrocoll..

[33] Tian H., Xu G., Yang B., Guo G. (2011). Microstructure and mechanical properties of soy protein/agar blend films: Effect of composition and processing methods. J. Food Eng..

[34] Wang X., Hu L., Li C., Gan L., He M., He X., Tian W., Li M., Xu L., Li Y. (2016). Improvement in physical and biological properties of chitosan/soy protein films by surface grafted heparin. Int. J. Biol. Macromol..

[35] Li K., Chen H., Li Y., Li J., He J. (2015). Endogenous cu and zn nanocluster-regulated soy protein isolate films: Excellent hydrophobicity and flexibility. RSC Adv..

[36] Otoni C.G., Avena-Bustillos R.J., Olsen C.W., Bilbao-Sáinz C., McHugh T.H. (2016). Mechanical and water barrier properties of isolated soy protein composite edible films as affected by carvacrol and cinnamaldehyde micro and nanoemulsions. Food Hydrocoll..

[37] Li K., Jin S., Chen H., He J., Li J. (2017). A high-performance soy protein isolate-based nanocomposite film modified with microcrystalline cellulose and cu and zn nanoclusters. Polymers.

[38] Li K., Jin S., Liu X., Chen H., He J., Li J. (2017). Preparation and characterization of chitosan/soy protein isolate nanocomposite film reinforced by cu nanoclusters. Polymers.

